# The emerging roles of long non-coding RNA (lncRNA) H19 in gynecologic cancers

**DOI:** 10.1186/s12885-023-11743-z

**Published:** 2024-01-02

**Authors:** Majid Ghasemian, Mojtaba Zehtabi, Mahrokh Abouali Gale Dari, Fatemeh Khojasteh Pour, Ghasem Azizi Tabesh, Farideh Moramezi, Razieh Mohammad Jafari, Mojgan Barati, Shahab Uddin, Maryam Farzaneh

**Affiliations:** 1 Department of Clinical Biochemistry, School of Medicine, Ahvaz Jundishapur University of Medical Sciences, Ahvaz, Iran; 2 Hematology and Oncology Research Center, Tabriz University of Medical Sciences, Tabriz, Iran; 3 Department of Obstetrics and Gynecology, School of Medicine, Ahvaz Jundishapur University of Medical Sciences, Ahvaz, Iran; 4 Department of Obstetrics and Gynecology, School of Medicine, Hamadan University of Medical Sciences, Hamadan, Iran; 5 Genomic Research Center, Shahid Beheshti University of Medical Sciences, Tehran, Iran; 6 Fertility, Infertility and Perinatology Research Center, Ahvaz Jundishapur University of Medical Sciences, Ahvaz, Iran; 7 Translational Institute and Dermatology Institute, Academic Health System, Hamad Medical Corporation, Doha, Qatar; 8 Department of Biosciences, Integral University, Lucknow, Uttar Pradesh 22602 India

**Keywords:** LncRNAs, H19, Gynecologic cancer, Tumorigenesis, Biomarker

## Abstract

Long non-coding RNA (lncRNA) H19 has gained significant recognition as a pivotal contributor to the initiation and advancement of gynecologic cancers, encompassing ovarian, endometrial, cervical, and breast cancers. H19 exhibits a complex array of mechanisms, demonstrating dualistic effects on tumorigenesis as it can function as both an oncogene and a tumor suppressor, contingent upon the specific context and type of cancer being investigated. In ovarian cancer, H19 promotes tumor growth, metastasis, and chemoresistance through modulation of key signaling pathways and interaction with microRNAs. Conversely, in endometrial cancer, H19 acts as a tumor suppressor by inhibiting proliferation, inducing apoptosis, and regulating epithelial-mesenchymal transition. Additionally, H19 has been implicated in cervical and breast cancers, where it influences cell proliferation, invasion, and immune evasion. Moreover, H19 has potential as a diagnostic and prognostic biomarker for gynecologic cancers, with its expression levels correlating with clinical parameters and patient outcomes. Understanding the functional roles of H19 in gynecologic cancers is crucial for the development of targeted therapeutic strategies and personalized treatment approaches. Further investigation into the intricate molecular mechanisms underlying H19’s involvement in gynecologic malignancies is warranted to fully unravel its therapeutic potential and clinical implications. This review aims to elucidate the functional roles of H19 in various gynecologic malignancies.

## Introduction

Gynecological cancers (GCs), encompassingbreast, cervical, ovarian, and uterine cancers, pose a substantial global health burden, with malignancies playing a prominent role [1]. These cancers affecting women’s health are a matter of great concern, as they have a significant impact on the lives of those affected [2, 3]. A variety of factors make the female population vulnerable to GCs, including genetic predisposition, lifestyle choices, exposure to certain viruses, and hormonal imbalances [4, 5]. Numerous studies have demonstrated a correlation between polycystic ovary syndrome (PCOS), a hormonal disorder marked by elevated androgen levels and the presence of numerous ovarian follicles, and endometrial cancer [6, 7]. Lifestyle factors are known to contribute significantly to GCs. For example, tobacco smoking has been linked to a higher risk of cervical cancer by accelerating the malignant transformation of cervical cells following human papillomavirus infection. Similarly, smoking has also been identified as a risk factor for the development of breast cancer. In contrast, the consumption of antioxidant vitamins has been suggested to interfere with these cancer-promoting effects [8, 9].

The current landscape of GC diagnosis and treatment embraces a multidisciplinary strategy that integrates diverse methodologies and technological advancements [10]. To evaluate the size and metastasis of GCs, imaging modalities such as ultrasound, computed tomography (CT), magnetic resonance imaging (MRI), and positron emission tomography (PET) scans are routinely employed [11]. Also, various biopsy techniques, including colposcopy [12], endometrial biopsy [13], hysteroscopy [14], and laparoscopy [15], are utilized to procure tissue samples from suspected tumors or abnormal regions for further analysis and confirmation. Molecular tests can identify specific biomarkers associated with GCs, such as human papillomavirus (HPV) testing for cervical cancer or genetic testing for ovarian cancer [16, 17]. The treatment of GCs depends on various factors including the type and stage of cancer [18], overall health of the patient, and the patient’s preferences [19]. Treatment options may include surgery to remove the cancerous tissue (laparoscopy, hysterectomy, oophorectomy) [20], radiation therapy using high-energy radiation beams to destroy cancer cells (externally or internally) [21], chemotherapy to kill cancer cells (orally or intravenously) in combination with surgery or radiation therapy [22], targeted drugs [23], and immunotherapy [24]. Over the years, treatment strategies for GCs have become more personalized and targeted [25]. In the case of ovarian cancer, the primary focus is on implementing tumor-reducing surgeries, followed by conventional platinum-based chemotherapy, which has been shown to be effective in many cases [26]. Similar to other types of cancer, the molecular features of GCs, particularly genomic analysis, are playing an increasingly significant role in the timely detection of these cancers and in the selection of appropriate therapeutic approaches [27, 28].

Current research has made it clear that despite being transcribed as RNAs, the majority of the human genome is not translated into proteins and instead plays various roles in the characteristics of cancers [29]. Among the different types of non-coding RNAs (ncRNAs), long ncRNAs (lncRNAs) are the most abundant subtype, consisting of linear RNAs that are longer than 200 nucleotides [30]. LncRNAs can be derived from various regions of the genome, including exons, introns, 5′ and 3′ untranslated regions, and intergenic regions [31]. Among the various lncRNAs that have been studied, H19 is one of the more extensively investigated ones. This particular lncRNA has been shown to play a significant role in cancer cell survival, progression, and metastasis, acting as either a promoter or a suppressor [32, 33]. The role of H19 in GCs has been a topic of intense research in recent years. Studies have shown that H19 contributes to the progression of ovarian and endometrial cancer through various mechanisms, such as inducing epithelial to mesenchymal transition (EMT). Conversely, the downregulation of H19 has been linked to increased proliferation of cervical cancer cells [34]. The focus of this review is on the potential functions of H19 in GCs.

## LncRNA H19: physiological and cancer-related properties

H19 is one of the most extensively studied classical lncRNAs due to its crucial roles in both normal developmental processes and pathological conditions such as cancer [35]. This RNA molecule is transcribed from the highly conserved H19 gene located at the 11p15.5 locus, and is approximately 2.3 kb in length. The H19 gene comprises of five exons and four introns and undergoes 3′ polyadenylation and 5′ capping. It is part of the H19/IGF2 cluster, in which the two genes exhibit imprinted features, with H19 transcribed from the maternal allele and Igf2 transcribed from the paternal allele [36]. The regulatory mechanisms underlying the expression of H19 gene products are complex and multifaceted. Furthermore, apart from its role as a lncRNA, the H19 gene exhibits an intriguing feature of encoding two miRNAs, specifically miR-675-3p and miR-675-5p. Extensive research has demonstrated the significant involvement of these miRNAs in orchestrating the intricate processes of skeletal muscle development and placental growth regulation [37, 38]. H19 has been shown to modulate gene expression through various mechanisms, including epigenetic remodeling and miRNA sponging [37]. The expression of this lncRNA is particularly high during tissue development in certain tissues, but its levels decrease after birth, with the notable exception of skeletal and cardiac muscle. H19 plays a crucial role in the differentiation of myoblasts by interacting with miR-106a and inhibiting its effects [36, 39]. H19 gene is involved in multiple machineries essential for development and normal in utero growth through DNA methylation [40]. H19 takes part in differentiation of stem cells residing human dental pulp [41]. Zeng et al. found that H19 knockdown leads to inhibition of human dental pulp stem cell differentiation; while its upregulation aids to drive this process. The activity of this lncRNA is mediated by S-adenosylhomocysteine hydrolase (SAHH) suppression, so called H19/SAHH axis, which inhibits distal-less homeobox 3 (DLX3) gene methylation resulting in positive regulation of odontogenic differentiation [42]. The role of H19 is also identified in angiogenesis at the mother-fetus interface. H19 is highly expressed in first-trimester trophoblasts of human specimens. In a study designed by Zeng et al. on HTR-8/SVneo, an extravillous trophoblast cell line, they discovered that overexpression of H19 led to cell migration and endothelial cell tube formation [42]. Whereas these events were decreased in H19-downregulated condition. The regulatory effect of H19 was exerted through H19/miR-106a-5p/VEGFA axis [43]. Moreover, H19 function as competing endogenous RNA (ceRNA) is evident in Wnt/β-catenin and SMAD-dependent pathways. In in vitro investigations, it was depicted that H19 has a pro-osteogenic effect in SMAD-dependent signaling by the means of TGF-β1/Smad3/HDAC pathway. Interestingly, H19 may cause in vivo bone formation [44]. Considering Wnt/β-catenin pathway, two anti-osteogenic miRNAs miR-22 and miR-141 sponging via H19 induces ALP, OCN, RUNX2 and BMP resulting in osteogenesis [45]. In the context of tension-induced osteogenesis, H19 also acts as a positive modulator by functioning as ceRNA for miR-138 in order to upregulate focal adhesion kinase (FAK), an essential item in osteogenesis mechano-transduction pathway [46]. Many studied lncRNAs are shown to be expressed significantly in human CNS indicating their remarkable effect in the brain development. H19 depicts a stable property in tissues evolving into the human CNS system (e.g. neural crest) [47]. These model statements depict the important effect of H19 in pathways related to normal histogenesis and development.

Along with the role of H19 in normal human development, there are pathologic conditions like cardiac muscle hypertrophy, osteoporosis and cancer that this lncRNA is known to take part [32, 48, 49]. H19 expression is often dysregulated in various types of cancer. It can act as an oncogene, promoting tumor growth, invasion, and metastasis. H19 can also interact with other genes and molecules to regulate key cancer-related processes [33]. Silencing of H19 results in cell death and progression arrest in cancers [50]. Mutations or abnormalities in the H19 gene or its regulatory regions can lead to developmental disorders, such as Beckwith-Wiedemann syndrome (BWS), characterized by overgrowth and increased cancer risk [51]. This lncRNA also can influence processes such as inflammation, endothelial dysfunction, and smooth muscle cell proliferation [52–54].

**Fig. 1:**
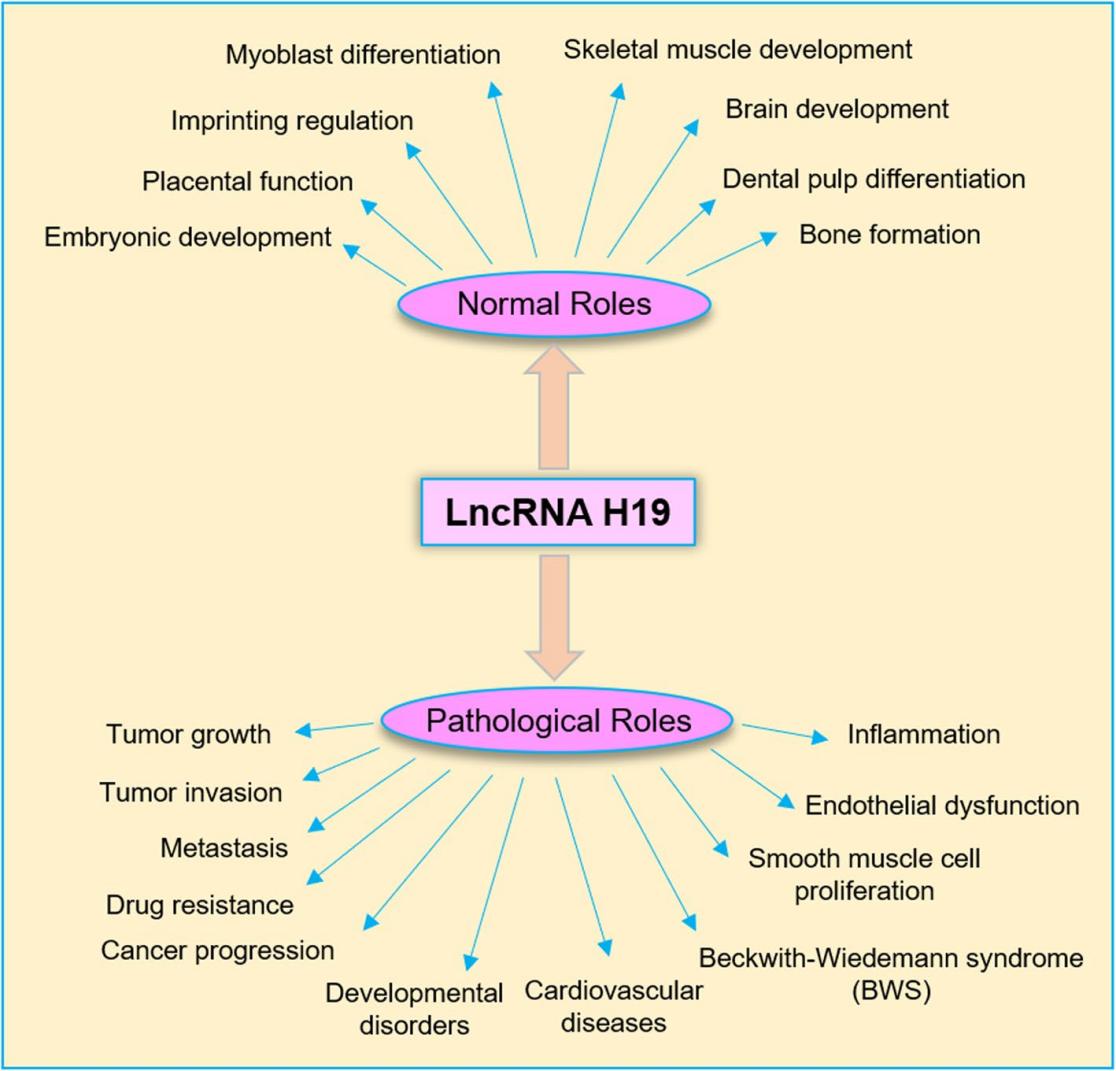
H19 exhibits a broad spectrum of functionality, intricately participating in a multitude of physiological processes, while also playing a significant role in the pathogenesis and advancement of diverse pathological conditions

In tumors, H19 has the ability to promote drug resistance by acting as a miR-200c sponge, leading to gefitinib resistance in non-small cell lung cancer (NSCLC). By reducing Bcl-2 expression and phosphorylated-Akt signaling, miR-200c sensitizes NSCLC tumor cells to gefitinib [55]. According to Yörüker et al., gastric cancer patients exhibit elevated levels of circulating H19 when compared to healthy samples, with a negative correlation observed between plasma H19 levels and tumor size. Additionally, the level of circulatory H19 was found to decrease following tumor removal [56]. The findings of this study highlight the diagnostic potential of H19 in cancer and suggest that it may serve as a promising prognostic marker for solid tumors. A meta-analysis performed by Liu et al. revealed that solid cancers with elevated H19 expression were linked to shorter overall and disease-free survival as well as advanced clinical stage [57]. In ovarian cancer, for instance, H19 has been found to play a role in promoting cell proliferation, invasion capability, and drug resistance [33]. Similarly, elevated levels of H19 expression have been observed in endometrial cancer, and these high levels have been linked to increased cell growth potential [58]. In a study conducted by Zhang et al., a positive correlation was found between H19 expression and estrogen and progesterone receptor levels, as well as lymph node metastasis, in BC patients, highlighting the oncogenic role of H19. Interestingly, the levels of H19 in the blood were observed to decrease following surgical excision of the tumor [59]. Aberrant expression of H19 in cervical cancer tissues has also been associated with cancer progression, providing another example of the oncogenic potential of this molecule [60]. In a research study on BC conducted by Smith et al., the level of HER2 was found to be higher in tumor tissues compared to normal tissues in the same patients. Moreover, the expression level of HER2 in the tumor tissues was found to be a potential predictor of disease progression and response to treatment [61]. Figure 1 provides the multifaceted functions of H19, encompassing both its normal physiological roles and its aberrant involvement in pathological conditions.

Given the significance of H19 in malignant diseases, particularly GCs, it would be beneficial to further investigate the molecular pathways involving H19 in GCs and analyze their impact on the advancement and outcome of these cancer subtypes.

## Exploring the significance of lncRNA H19 in gynecologic cancers: functional roles and molecular mechanisms

Multiple studies have established that H19 has the ability to regulate numerous miRNA/mRNA axes, thereby playing a crucial role in the development of GCs (as outlined in Table 1). In the following section, we have provided an overview of various H19-associated molecular pathways that are pivotal in the initiation and progression of GCs.

**Table 1:** Functional roles of lncRNA H19 in gynecologic cancers

Cancer Type	Object / Target		Results		Refs.
	Suppression	Stimulation	Increase	Decrease	
**Breast Cancer**		17β-estradiol	Estrogen-induced cell growth and proliferation		[62]
		ERα-H19-BIK/NOXA signaling		Apoptosis	[63]
	let-7	LIN28	Cancer stem cell viability		[64]
		CUL4A-ABCB1/ MDR1 and ABCC4/MRP4 pathway	Drug resistance		[65]
	miR-200b/c and let-7b		EMT potential by increasing GIT2 and CYTH3 expression		[66]
	ERα expression		Endocrine therapy resistance		[67]
		Wnt/β-catenin pathway	EMT	Apoptosis	[68]
		Akt signaling pathway	Paclitaxel resistance	Apoptosis	[69]
	SAHH/DNMT3B		Autophagy Tamoxifen resistance		[70]
	miR-675		Trastuzumab resistance TNM stage	Progression-free survival	[71]
	miR-491-5p	ZNF703	Cell proliferation Tumor invasion	Apoptosis	[72]
	miR-340-3p	YWHAZ	Cell proliferation Metastasis (via EMT induction)	Apoptosis	[73]
	PARP1		Doxorubicin resistance		[74]
	p53	TNFAIP8	Cell proliferation Metastasis (via EMT induction)	Cell cycle arrest	[75]
		N-acetyltransferase 1 promoter methylation	Tamoxifen resistance		[76]
	Lipid ROS	Beclin1 LC3 GSH content	Autophagy	Ferroptosis	[77]
**Cervical Cancer**	No mentioned object	No mentioned object	Anchorage-independent multicellular spheroid growth		[78]
	No mentioned object	No mentioned object		Relapse-free survival	[79]
	miR-138-5p		Tumor proliferation Lymph node metastasis FIGO stage	Tumor differentiation Overall survival	[80]
			Clinicopathological characteristic		[81]
**Ovarian Cancer**	Caspase-dependent pathway of apoptosis	Cyclin A2 and B1	Tumor cell growth G2/M transition	Apoptosis	[82]
		Glutathione metabolism (GSH content)	Cisplatin resistance	Relapse-free survival	[83]
	miR-370-3p		EMT induced by TGF-β		[84]
	p21 PTEN	EZH2	Cisplatin resistance	Apoptosis	[85]
	miR-324-5p	PKM2	Warburg effect/Tumor glucose consumption Tumor cell proliferation		[86]
	E-cadherin	Twist Slug Snail	EMT Cisplatin resistance		[87]
		IGF2			[88]
	miR-140	Wnt1	Cancer cell proliferation Colony formation and migration	Patient probability of survival	[89]
	E-cadherin	N-cadherin	Cancer cell viability, proliferation and invasion		[90]
**Endometrial Cancer & Endometriosis**	let-7	IGF1R Ki67 PCNA	Cancer cell proliferation		[91]
	let-7	Hmga2 c-Myc Imp3	Cancer cell invasion and migration		[92]
	E-cadherin	Snail	EMT and cancer progression		[93]
	miR-612	HOXA10	Cancer cell viability	Overall survival	[58]
	miR-342-3p	IER3		Th17 differentiation Endometrial stromal cell proliferation	[94]
	miR-124-3p	ITGB3	Ectopic endometrial cell proliferation and invasion		[95]
	miR-216a-5p	ACTA2	Endometrial stromal cell migration and invasion		[96]
	No mentioned object	No mentioned object	Infertility Recurrence Bilateral ovarian lesions rAFS stage CA125 level		[61]
	No mentioned object	No mentioned object	Tumorigenicity of ectopic endometrial cells		[97]
	No mentioned object	No mentioned object	Proliferation and migration of ectopic endometrial stromal cell		[98]
		IGF1	Cell proliferation and differentiation		[99]
	No mentioned object	No mentioned object	Clinical stage(rASRM classification scoring)		[100]

### Breast cancer

**Fig. 2:**
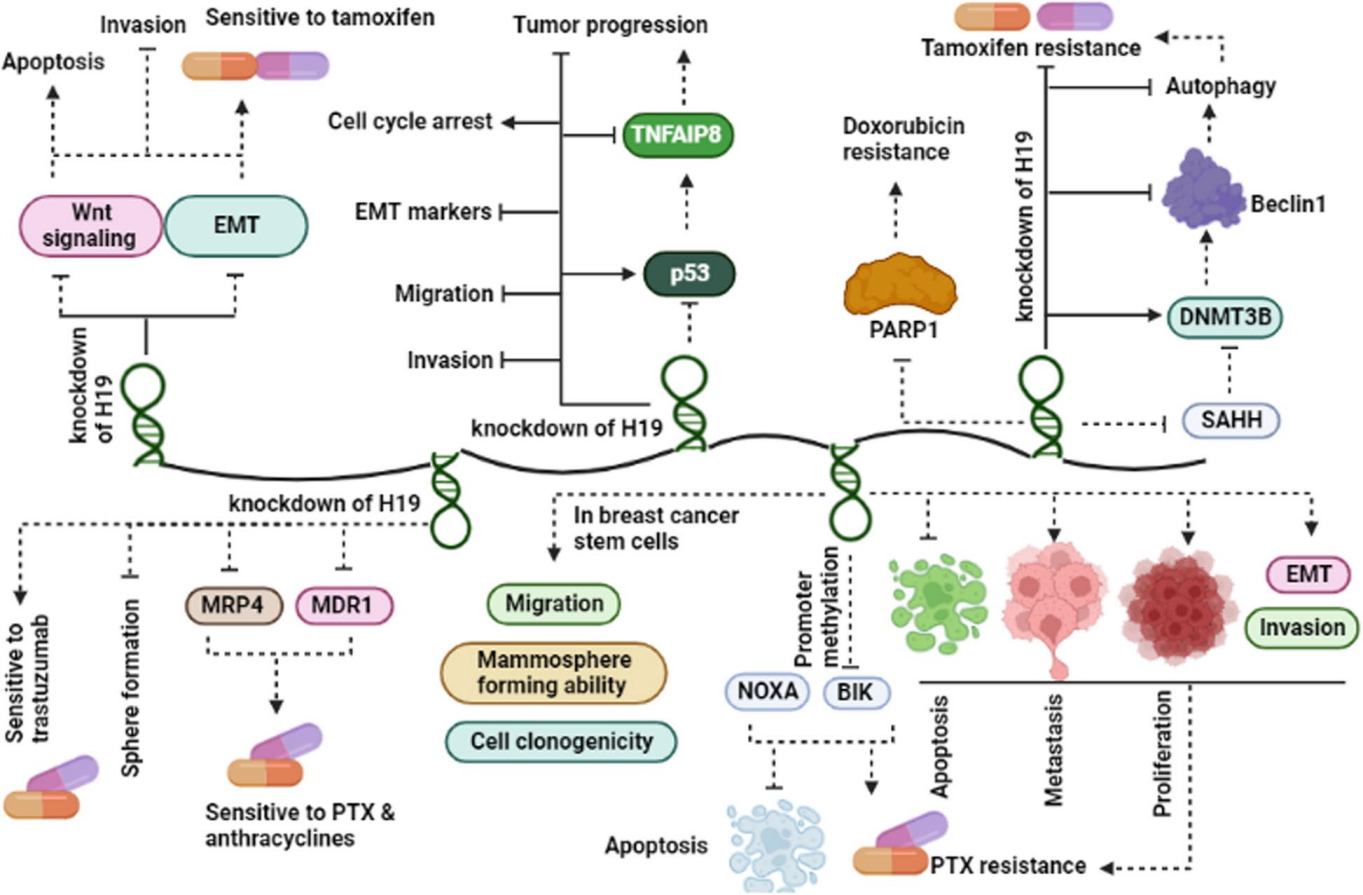
The Involvement of H19 in Breast Cancer Progression: Mechanisms and Implications. H19 is involved in drug resistance, invasion, migration, and apoptosis through various mechanisms. PTX: paclitaxel, EMT: Epithelial-mesenchymal transition, SAHH: S–adenosyl homocysteine hydrolase

Studies have shown that H19 expression levels are higher in ER-positive tumor tissues than in ER-negative ones, and that 17β-estradiol can increase H19 expression in MCF-7 cells, which are ER-positive. Thus, it can be inferred that H19 is an estrogen-inducible gene that plays a critical role in the survival of MCF-7 cancerous cells [62]. The role of H19 in drug resistance is crucial, as it has been shown to inhibit the pro-apoptotic factors BIK and NOXA, resulting in a decreased rate of apoptotic cell death in response to paclitaxel (PTX). H19 achieves this effect through promoter methylation of BIK [63]. Peng et al. demonstrated a significant increase in H19 expression levels in breast cancer stem cells (BCSCs). Furthermore, they found that overexpression of H19 significantly enhanced mammosphere formation ability, migration, and clonogenicity of BC cells [64]. MRP4 and MDR are the pivotal molecules responsible for conferring resistance to H19 in cancer cells. Upon silencing of the H19 lncRNA in doxorubicin-resistant MCF-7 cells, there was a marked improvement in the sensitivity of cancer cells to chemotherapy agents such as paclitaxel and anthracyclines, accompanied by a significant decrease in the expression levels of MRP4 and MDR1 [65]. Resistance to endocrine therapy is a defining characteristic of ER+ BC. Pratima et al. demonstrated that following treatment of endocrine-resistant BC cells with Fulvestrant and Tamoxifen, there was a significant upregulation in the expression levels of the H19. The expression of H19 is regulated by the c-MET and Notch signaling pathways, and inhibition of these pathways was shown to decrease H19 expression, leading to an increase in the sensitivity of endocrine-resistant cells to Fulvestrant and Tamoxifen [67]. A different research revealed that inhibiting the EMT and Wnt signaling pathway through knockdown of H19 not only enhances the rate of apoptosis and sensitivity to tamoxifen but also reduces the invasiveness of cancer cells [68]. Han et al. demonstrated that silencing of H19 enhanced the chemosensitivity of triple-negative breast cancer (TNBC) cell lines to PTX by promoting apoptosis through the regulation of the Akt signaling pathway [69]. Numerous studies have confirmed that aldehyde dehydrogenase 1 (ALDH1) is a well-established marker of stem cells. Shima et al. reported a strong association between ALDH1 positivity and H19 expression in BC patients. They also demonstrated that the silencing of H19 significantly decreased the ability of cancer cells to form spheres, indicating a potential role for H19 in regulating the stemness of BC cells [101]. The involvement of H19 in tamoxifen resistance in BC cell lines has been attributed to its interaction with SAHH, which decreases DNMT3B expression and promotes Beclin1, subsequently enhancing the autophagy process. Knockdown of H19 has been shown to reduce tamoxifen resistance by increasing the methylation of the Beclin1 promoter by DNMT3B [70]. In trastuzumab-resistant BC cells, there is a notable upregulation in the expression levels of H19. However, studies have shown that silencing of H19 can effectively overcome trastuzumab resistance in these cells [71]. H19 has been shown to significantly promote invasion, metastasis, cell proliferation, and EMT process while inhibiting apoptosis in PTX-resistant BC cells. Conversely, silencing of H19 has been demonstrated to have the opposite biological effects, suggesting a potential role for H19 in the development and progression of PTX-resistant BC [73]. The involvement of H19 in doxorubicin resistance has been observed in both tissue and cancerous cell lines. H19 promoted drug resistance by downregulating PARP1, a key regulator of DNA repair, highlighting the potential role of H19 in modulating cellular responses to chemotherapy agents [74]. NFAIP8, a target gene of p53 and a key player in the progression of cancer through various mechanisms such as EMT, is upregulated by H19 through its antagonistic effect on p53. Silencing of either TNFAIP8 or H19 has been shown to reduce the proliferation, invasion, migration, and expression of EMT markers, while increasing the arrest of the cell cycle. These findings suggest that the H19/p53/TNFAIP8 axis plays a critical role in tumor progression mediated by the upregulation of TNFAIP8 through the inhibition of p53 by H19 [75]. Figure 2 illustrates the various mechanisms by which H19 contributes to the progression of BC.

### Ovarian cancer

**Fig. 3:**
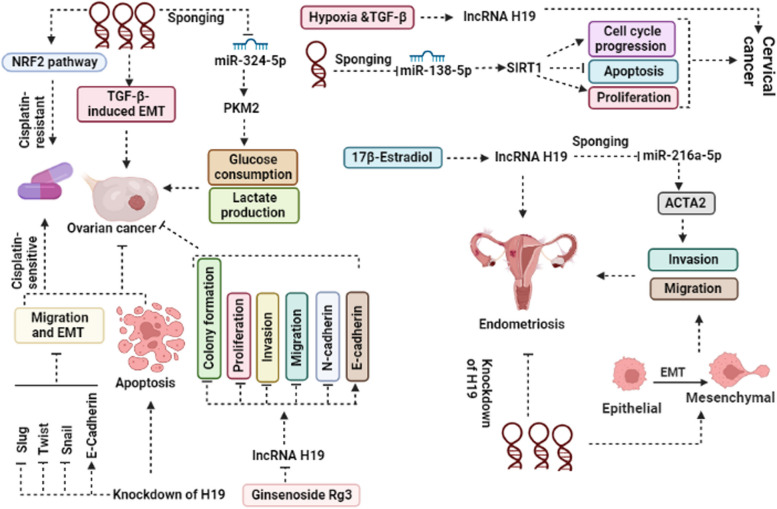
The Mechanisms of H19 in Regulating Tumor Cell Behavior in Ovarian Cancer, Cervical Cancer, and Endometriosis. H19 plays a crucial role in promoting tumor progression through various mechanisms, including apoptosis inhibition, promotion of epithelial-to-mesenchymal transition (EMT), tumor cell invasion, migration, and proliferation. Of these mechanisms, the activation of EMT is particularly pivotal in promoting tumorigenesis via H19

H19 has been found to play a role in the development of ovarian cancer by regulating various pathways, as evidenced by increased expression levels in cisplatin-resistant A2780-DR cells. Further investigations, both in vitro and in vivo, have shown that knockdown of H19 leads to increased sensitivity of A2780-DR cells to cisplatin and reduced expression of NRF2-targeted proteins, including G6PD, GSR, NQO1, GCLM, GSTP1, and GCLC. Given its involvement in the glutathione metabolism pathway, it can be concluded that H19 contributes to drug resistance by modulating glutathione metabolism [83]. Li et al. has demonstrated that H19 plays a crucial role in TGF-β-induced EMT, and that this function is exerted through the sponging of miR-370-3p by the H19 [84]. The anti-seizure drug valproic acid has been shown to decrease the expression of H19 in the cisplatin-resistant ovarian cell line A2780/CP. Subsequent silencing of H19 led to a significant increase in both the rate of apoptosis and sensitivity to cisplatin in the A2780/CP cell line [85]. The modulation of glycolysis metabolism by H19 has been shown to regulate the Warburg effect, as reported by Zheng et al. Specifically, silencing of H19 resulted in decreased glucose consumption, PKM2 expression, and lactate production. This function was found to be exerted through the sponging of miR-324-5p by H19 [86]. In the cisplatin-resistant ovarian cancer cell line OVCAR3/DDP, the expression level of H19 was found to be significantly increased compared to the OVCAR3 cell line. Additionally, OVCAR3/DDP cells exhibited upregulation of EMT markers such as slug, twist, and snail, and decreased E-Cadherin levels. Subsequent knockdown of H19 in OVCAR3/DDP cells led to a suppression of migration and EMT-positive markers, while promoting E-Cadherin expression [87]. Recent reports suggest that Ginsenoside Rg3, a tetracyclic triterpenoid, has the ability to inhibit proliferation, invasion, migration, and colony formation of ovarian cancer cells. Additionally, treatment with Ginsenoside Rg3 was found to decrease the expression of N-cadherin while increasing E-cadherin levels, and these effects were attributed to the suppression of H19 [90]. Figure 3 shows different signaling pathways associated with H19 that impact ovarian cancer.

### Cervical cancer

The expression level of H19 was found to be modulated under conditions of both TGF-β and hypoxia. H19 has the ability to enhance both independent growth and anchorage-specific growth of cervical cancer cell lines [78]. Previous studies have indicated that SIRT1 plays a critical role in various biological processes, including cancer progression. Lei et al. have demonstrated that SIRT1 is a direct target of miR-138-5p in ovarian cancer, and that overexpression of miR-138-5p results in G0/G1 cell cycle arrest, induction of apoptosis, and inhibition of cell proliferation. However, in contrast, H19 has been shown to upregulate SIRT1 and promote cervical cancer progression via sponging of miR-138-5p [80]. Another study has reported that silencing of H19 results in decreased cell proliferation, induction of apoptosis, and cell cycle arrest in the cervical cancer cell lines OV90 and SKOV3 [82]. Figure 3 displays various H19-related signaling pathways that have an impact on cervical cancer.

### Endometrial cancer

The role of H19 in the progression of endometriosis has been the subject of conflicting findings in previous studies. While some investigations have reported upregulation of H19 expression in tumoral tissue compared to control, others have reported the opposite results. Sanaz et al. reported that the expression level of H19 in patients with eutopic endometrium was significantly lower than that of normal controls [91]. Another study has shown that H19 plays a role in the progression of endometrial cancer by affecting the EMT process. Silencing of H19 was found to reduce invasion and migration in the HEC-1-B cancer cell line, while also leading to a decrease in Snail expression and an increase in E-cadherin expression. Based on these findings, it can be concluded that H19 promotes endometriosis by modulating the EMT process [93]. It was found that the expression of H19 was significantly suppressed in mononuclear cells obtained from peritoneal fluid of patients with endometriosis [94]. It has been reported that H19 can stimulate the formation of fibrotic tissue in women suffering from endometriosis through the H19/miR-216a-5p/ACTA2 pathway. miR-216a-5p can bind to H19 and to ACTA2 in the 3′ untranslated region (3′UTR) [96]. In vivo investigations using a nude mouse model have demonstrated that silencing of H19 leads to a significant decrease in endometriosis [97]. It has been observed that 17β-estradiol plays a role in regulating the expression pattern and function of H19 in patients with endometriosis. Based on these findings, it can be concluded that 17β-estradiol is involved in the development of endometriosis through the regulation of H19 [98]. According to a study by Sedigheh et al., the expression level of H19 was found to be significantly decreased in both ectopic and eutopic endometrial tissues compared to control tissues. The researchers suggested that H19, through downregulation of IGFI and IGFII, leads to impairment of cellular growth regulation and differentiation [99]. Figure 3 presents different signaling pathways associated with H19 that exert an impact on endometrial cancer.

### The ceRNA network of H9 in gynecologic cancers

**Fig. 4:**
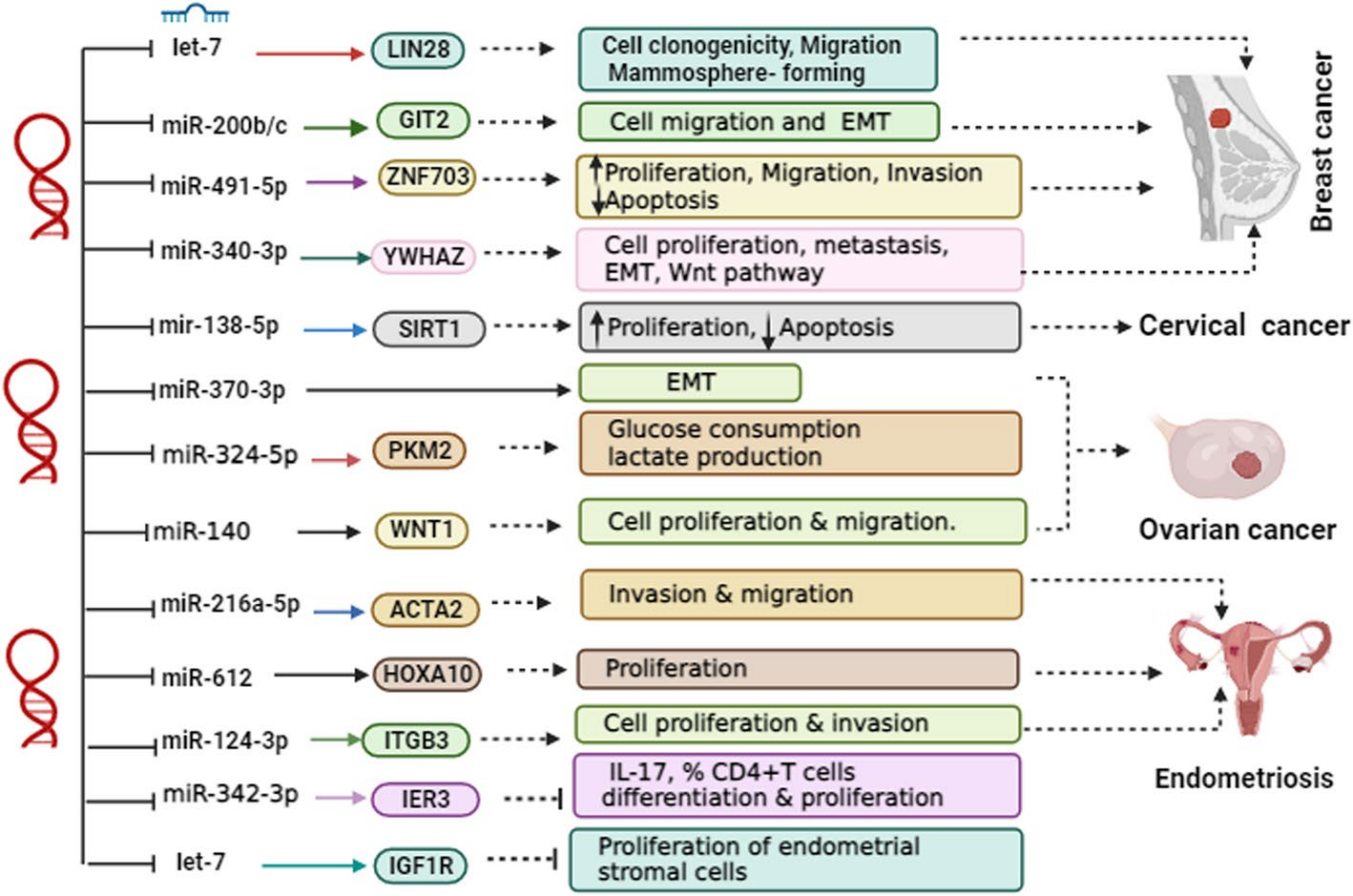
The Functional Impact of H19-Mediated Gene Regulation on Downstream Targets. Various H19-associated miRNAs have been identified as playing a critical role in the pathogenesis of gynecologic cancers

Several studies have highlighted the role of lncRNAs as ceRNAs that sequester specific miRNAs, thereby diminishing their regulatory impact on target mRNA genes. This interaction between miRNAs and lncRNAs plays a significant role in the development and progression of GCs. Notably, H19 has been identified as a key participant in miRNA sponging within GCs, including miRNA let-7 [64], miR-200b/c [66], miR-491-5p [72], miR-340-3p [73], miR-138-5p [80], miR-370-3p [84], miR-324-5p [86], miR-140 [89], let-7 [91], miR-612 [58], miR-342-3p [94], miR-124-3p [95], and miR-216a-5p [96]. H19 functions as a ceRNA by sequestering let-7, thereby upregulating LIN28 as a direct target of let-7. Conversely, the upregulation of LIN28 suppresses let-7 biogenesis, establishing a reciprocal negative feedback loop involving H19, let-7, and LIN28 as a transcriptional factor [64]. In addition, Wu et al. reported that H19, through its interaction with miR-200b/c and let-7b, modulates the expression of its target genes GIT2 and CYTH3, respectively, which encode regulators of the RAS superfamily. This modulation promotes cell migration and EMT in tumor cells [66]. Also, H19 negatively regulated miR-491-5p expression and inversely modulated ZNF703 expression as a target of miR-491-5p. Therefore H19/miR-491-5p/ZNF703 axis has a critical role in development of BC [72]. Furthermore, H19 has been shown to regulate the expression of miR-491-5p and inversely modulate ZNF703, playing a critical role in breast cancer development [73]. In cervical cancer, H19 acts as a miR-138-5p sponge, reducing SIRT1 expression as a miR-138-5p target gene [80]. Moreover, H19 is involved in the TGF-β/H19/miR-370-3p/EMT signaling axis in ovarian cancer progression [84]. In ovarian cancer, H19 regulates the Warburg effect by directly binding to miR-324-5p and upregulating PKM2, a target of miR-324-5p, thereby enhancing glycolysis metabolism [86]. The H19/miR-140/Wnt1 axis is pivotal in promoting proliferation and migration in ovarian cancer [89]. In endometrial stromal cells, the H19/let-7/IGF1R axis is involved in impaired endometrial proliferation [91]. Furthermore, H19 regulates the percentage of Th17 cells/CD4+ T cells and IL-17 levels via the miR-342-3p/IER3 axis [58]. Elevated H19 levels in ectopic endometrial cells contribute to cell proliferation and invasion through modulation of miR-124-3p and ITGB3 [94]. In women with endometriosis, ectopic endometrial cells have an increasing level of H19. Cell proliferation and invasion was inhibited after H19 down-regulation. H19 exerts this function by modulating miR-124-3p and ITGB3 [95]. Additionally, estrogen-induced alterations in stromal cell invasion and migration in endometriosis are mediated by the H19/miR-216a-5p/ACTA2 axis [96]. Figure 4 provides an overview of the crucial H19-miRNAs involved in the pathogenesis of GCs.

### Clinical application of H19 in gynecologic cancers

The successful translation of laboratory-based and pre-clinical findings in biology and molecular research to a clinical context plays a pivotal role in advancing cancer research. This critical process necessitates the modification and adaptation of experimental results to render them applicable in clinical settings. Ultimately, the effective translation of basic research findings to clinical practice is imperative for enhancing our comprehension of gynecologic cancers and devising efficacious therapeutic approaches to benefit patients [102]. It was found that the expression levels of H19 were significantly elevated in atypical in situ breast lesions within a Lebanese population, surpassing those in non-malignant lesions. Notably, the highest levels of H19 were detected in malignant tissues. These compelling findings indicate that H19 has the potential to serve as a promising biomarker for the early detection of BC [103]. The clinical significance of H19 levels has been investigated in plasma liquid biopsies, an emerging and dynamic field of research due to its non-invasive and easily accessible nature. Zhang et al., illustrated that the levels of H19 were markedly elevated in the serum of BC patients compared to healthy individuals. Additionally, increased levels of circulating H19 were found to be correlated with hormone receptor expression and lymph node metastasis. The study also determined a specificity of plasma H19 levels for BC exceeding 0.85, emphasizing its potential as a reliable biomarker for BC [59]. It was shown that pre-therapeutic serum levels of H19 could serve as a predictive indicator of neoadjuvant treatment outcomes in BC patients. Notably, patients with lower levels of serum H19 were shown to have a higher probability of achieving a complete pathological response to the treatment. Interestingly, patients with elevated plasma H19 levels were found to exhibit the triple-negative BC subtype. These significant findings suggest that H19 holds promise as a valuable biomarker for predicting treatment response and identifying specific disease subtypes in BC patients [104]. In the context of TNBC, increased levels of H19 have been linked to reduced overall survival and disease-free survival durations. Furthermore, a separate study unveiled an association between the rs2107425 single nucleotide polymorphism (SNP) located near H19 and decreased metastasis-free survival. Moreover, serum levels of H19 have been demonstrated to decline following mastectomy, indicating the potential clinical value of H19 in assessing the efficacy of surgical interventions [32, 105]. Zhang et al. examined the relationship between genetic variations in the IGF2/H19 gene locus and the risk of developing epithelial ovarian cancer (EOC) in a Chinese population. Their findings unveiled a noteworthy connection, indicating that three specific polymorphisms, namely rs3741206, rs2525885, and rs2839698, were significantly associated with an elevated susceptibility to EOC, particularly in patients aged 47 years and older. Moreover, within the rs2525885 subgroup, individuals possessing the TC + CC genotype demonstrated a higher likelihood of advanced FIGO stage, implying that this particular polymorphism may not only contribute to the initiation of cancer but also exert influence on the progression of tumors [88]. Higher levels of H19 expression in tumor tissues among EOC patients were found to be linked with shorter overall survival, indicating its potential as a prognostic marker. Moreover, H19 expression was identified as a predictive factor for carboplatin resistance in EOC cells, suggesting its involvement in the development of chemoresistance and unfavorable treatment outcomes in this disease [106]. The impact of IGF2/H19 gene polymorphisms on platinum resistance has also been investigated in EOC patients. A study by Zeng et al. revealed that individuals with the rs4244809 GG genotype exhibited increased sensitivity to platinum-based chemotherapy agents [107]. Additionally, serum H19 has emerged as a potential biomarker for the diagnosis and monitoring of cervical cancer. Zhao et al. observed elevated levels of serum H19 in cervical cancer patients compared to healthy individuals, with a significant reduction after surgery. Serum H19 exhibited a specificity of nearly 95% in diagnosing cervical cancer, although its sensitivity was relatively low at 30.8%. Notably, plasma H19 levels did not show an association with tumor stage [108]. H19 gene polymorphisms were found to have predictive value for clinicopathological features in cervical cancer patients. In a study involving a Tai population, patients with CC/CT genotypes in H19 rs2839698 demonstrated a lower risk of pelvic node metastasis compared to those with the TT genotype. Similarly, patients with AA/AG genotypes in rs3741219 exhibited a lower risk of pelvic lymph node metastasis, as well as lower rates of parametrium and vaginal invasion, compared to those with the GG genotype. However, no statistically significant differences in prognostic parameters were observed among the various SNP polymorphisms [81]. According to Liu et al., both eutopic and ectopic endometrial tissues in endometriosis patients exhibited upregulated H19 expression, which positively correlated with disease recurrence, infertility, bilateral ovarian lesions, and the revised American Fertility Society (rAFS) stage. Their study demonstrated that high ectopic H19 expression had a sensitivity of 90.9% in predicting disease recurrence [61]. However, in contrast to these findings, Szaflik et al. reported decreased levels of H19 expression in endometrial tissues of endometriosis patients compared to healthy controls. Furthermore, among endometriosis patients, there was an association between H19 expression and the rAFS score for reproductive medicine classification of endometriosis (rASRM) score [100].

Despite the remarkable discoveries regarding the utilization of tissue/serum H19 levels in assessing the clinical characteristics of GC patients, there remains a need for further investigation to establish the expression level or specific single nucleotide polymorphisms (SNPs) of H19 as a suitable biomarker. One limitation is the current scarcity and heterogeneity of the studied SNPs, which necessitates their validation in larger populations. Additionally, certain studies have focused on restricted populations where the expression level of H19 could be influenced by various confounding factors. The role of H19 in endometriosis, as revealed by both clinical and basic studies, presents contradictory data, highlighting the need for additional confirmation. Furthermore, the availability of studies assessing sensitivity and specificity is limited, and they employ diverse sources such as tissue and serum samples. Moreover, some reported ratios exhibit low values and lack potential reliability, thereby making false results unavoidable without further confirmatory research.

## Conclusion

This review study extensively examines the roles of H19 in the development of GCs. Based on the comprehensive collection of molecular and clinical studies discussed herein, H19 emerges as a noteworthy molecular determinant in the landscape of GC tumor initiation and progression. The involvement of H19 in diverse and distinctive pathways holds promise for researchers seeking to unravel the intricate molecular interactions underlying GC pathogenesis. However, further clinical investigations are warranted to explore additional potential molecular markers in conjunction with H19, in order to obtain a more comprehensive understanding of the disease. A significant challenge in the treatment of GCs is drug resistance, and considering the existing data, targeting H19 may offer a promising approach to mitigate this burden. Although the aforementioned models show promising potential for clinical use of H19 expression as a biomarker in either tumor tissue or plasma samples for disease detection and prognosis prediction in GCs, Further research is required to fully establish H19 as a dependable and consistent biomarker. Integrating the measurement of H19 levels in tumor tissue and/or plasma, along with the analysis of its specific SNPs, in conjunction with established modalities such as molecular tests and imaging characteristics, has the potential to enhance the precision and reliability of diagnostic tests, thereby warranting further investigation. Furthermore, conducting specific in vivo studies to evaluate the potential of H19 as a therapeutic target for reducing the occurrence of therapy resistance represents a promising avenue in GC research. Deliberate manipulation of H19 expression levels within tumor cells could also serve as a means to modulate their aggressive behavior. Furthermore, larger sample size studies involving diverse ethnicities, different stages of disease, and varying responses to therapy would be necessary to establish the value of H19 as a diagnostic, prognostic, or therapeutic biomarker.

## Data Availability

The datasets used and/or analyzed during the current study are available from the corresponding author on reasonable request.

## Abbreviations

lncRNAs: long non-coding RNAs
GCs: gynecological cancers
PCOS: polycystic ovary syndrome
ncRNAs: non-coding RNAs
EMT: epithelial to mesenchymal transition
SAHH: S-adenosylhomocysteine hydrolase
DLX3: distal-less homeobox 3
ceRNA: endogenous RNA
FAK: focal adhesion kinase
NSCLC: non-small cell lung cancer
PTX: paclitaxel
BCSCs: breast cancer stem cells
ALDH1: aldehyde dehydrogenase 1
EOC: epithelial ovarian cancer
rAFS: revised American Fertility Society.
